# Examination of mitochondrial function and synaptic proteins in male and female Alzheimer's disease postmortem brain

**DOI:** 10.1002/alz70855_105880

**Published:** 2025-12-24

**Authors:** Alexander J.T. Yang, Ahmad Mohammad, Robert W.E. Crozier, Lucas Maddalena, Evangelina Tsiani, Adam J MacNeil, Gaynor Spencer, Paula Duarte‐Guterman, Jeffery Stuart, Rebecca EK MacPherson

**Affiliations:** ^1^ University of Kansas Medical Center, Kansas City, KS, USA; ^2^ University of Toronto, Toronto, ON, Canada; ^3^ Brock University, St Catharines, ON, Canada; ^4^ Centre for Neuroscience ‐ Brock University, St Catharines, ON, Canada; ^5^ Brock University, St. Catharines, ON, Canada; ^6^ Brock University, Department of Health Sciences, St. Catharines, ON, Canada; ^7^ Brock University, Centre for Neurosciences, St. Catharines, ON, Canada

## Abstract

**Background:**

Inflammation and mitochondrial impairments, driven by metabolic dysfunction, have been suggested to underly the synaptic damage seen with Alzheimer's disease. This study examined if human Alzheimer's disease brains have significant differences in metabolic, synaptic, and inflammatory markers compared to aged‐matched control brains. Sex‐specific differences were also examined as post‐menopausal females disproportionally present with AD compared to age‐matched males.

**Methods:**

Prefrontal cortex samples were taken from post‐mortem age‐matched female and male donors with/without Alzheimer's disease (*n* = 10 per sex and condition). Samples were prepared and analysed by immunofluorescence, western blot, ELISA‐based inflammatory cytokine panels, and mitochondrial respiration analysis.

**Results:**

Both AD males and females had higher Akt phosphorylation (*p* = 0.0064) but only AD males had higher downstream mTOR phosphorylation (*p* = 0.0363). AD samples had greater mitochondrial complex III (*p* = 0.0186), and V (*p* = 0.0341) content compared to controls while control males had less complex IV than control females (*p* = 0.0105). No difference in complex I respiration was found for any group, but AD females had lower Complex IV respiration compared to female controls (*p* = 0.0013). AD individuals had greater expression of synaptic markers AMPA GluA1 (*p* = 0.0256) and synaptophysin (*p* = 0.0432). Only AD females had a higher expression in the synaptic marker ELKS1 in white matter (*p* = 0.0116). Only the cytokine IL‐2 was found to be greater in AD individuals (*p* = 0.0390). Total Iba‐1 content was different between groups in both gray and white matter where Iba‐1 was lower in both AD females and males in gray matter (*p* = 0.0296), and AD females had higher Iba‐1 compared to all other groups in white matter (*p* = 0.0245). Microglia were then assigned as having either ameboid, hypertrophic, dystrophic, ramified, or rod‐like morphology. Rod‐like microglia were lower in the grey matter of AD samples (*p* = 0.0345) and there were no differences by sex.

**Conclusion:**

We demonstrate differences in Akt signaling and mitochondrial content in PFC samples from AD donors. Additionally, this study shows greater changes in metabolic signaling in females than in males, and that this dysregulation in signaling with AD development is unique to females when compared to males.

